# Low genetic diversity and potential inbreeding in an isolated population of alder buckthorn (*Frangula alnus*) following a founder effect

**DOI:** 10.1038/s41598-017-03166-1

**Published:** 2017-06-07

**Authors:** Caroline M. V. Finlay, Caroline R. Bradley, S. Jane Preston, Jim Provan

**Affiliations:** 1 School of Biological Sciences, Queen’s University Belfast, Belfast, BT9 7BL UK; 2 Institute of Biological, Environmental and Rural Sciences, Aberystwyth University, Aberystwyth, SY23 3DA UK; 3 ATECNI Environmental Consultancy, 31 Castlewellan Road, Banbridge, BT32 4JQ UK

## Abstract

Alder buckthorn (*Frangula alnus*) is one of Ireland’s rarest tree species, and in Northern Ireland the species is now restricted to a single population in Peatlands Park, Co. Armagh numbering *ca*. 140 mature trees. Genotyping of 95% of the trees at nine nuclear microsatellite loci revealed that levels of genetic diversity within this population were generally lower than those reported from larger populations in Spain. Analysis of six chloroplast microsatellite loci revealed no variation. The level of *F*_*IS*_ was significantly higher than that in the Spanish populations, as well as in other populations across Europe, potentially indicating inbreeding. Spatial autocorrelation analysis indicated some evidence of fine-scale genetic structuring, most likely due to limited seed dispersal, but the overall level of differentiation between subpopulations was low, indicating high levels of gene flow, probably due to cross-pollination by bees. Our results are consistent with a gradual population expansion from a limited number of individuals. We suggest that more immediate conservation efforts might be best focused on ensuring suitable habitat for the continued recovery of this isolated population.

## Introduction

Populations of endangered or threatened species tend to be small and/or isolated and are thus particularly vulnerable to stochastic processes. These problems are further exacerbated at the genetic level, where the increased effects of genetic drift and potential for inbreeding can lead to low levels of genetic variation^1, 2^. This can be further compounded if such populations have been founded by a limited number of individuals^3^, since genetically depauperate populations tend to have reduced evolutionary potential, which can increase the risk of extinction^4, 5^. Where populations are fragmented, as is often the case in threatened taxa, reduced levels of gene flow between fragments can also aggravate the problems associated with limited genetic diversity, as there is less scope for immigration of alleles to counter the effects of drift^6, 7^. Consequently, knowledge of the levels and patterns of genetic diversity in populations of threatened species are vital to the formation of well-informed, effective conservation plans^8, 9^.

**Figure 1:**
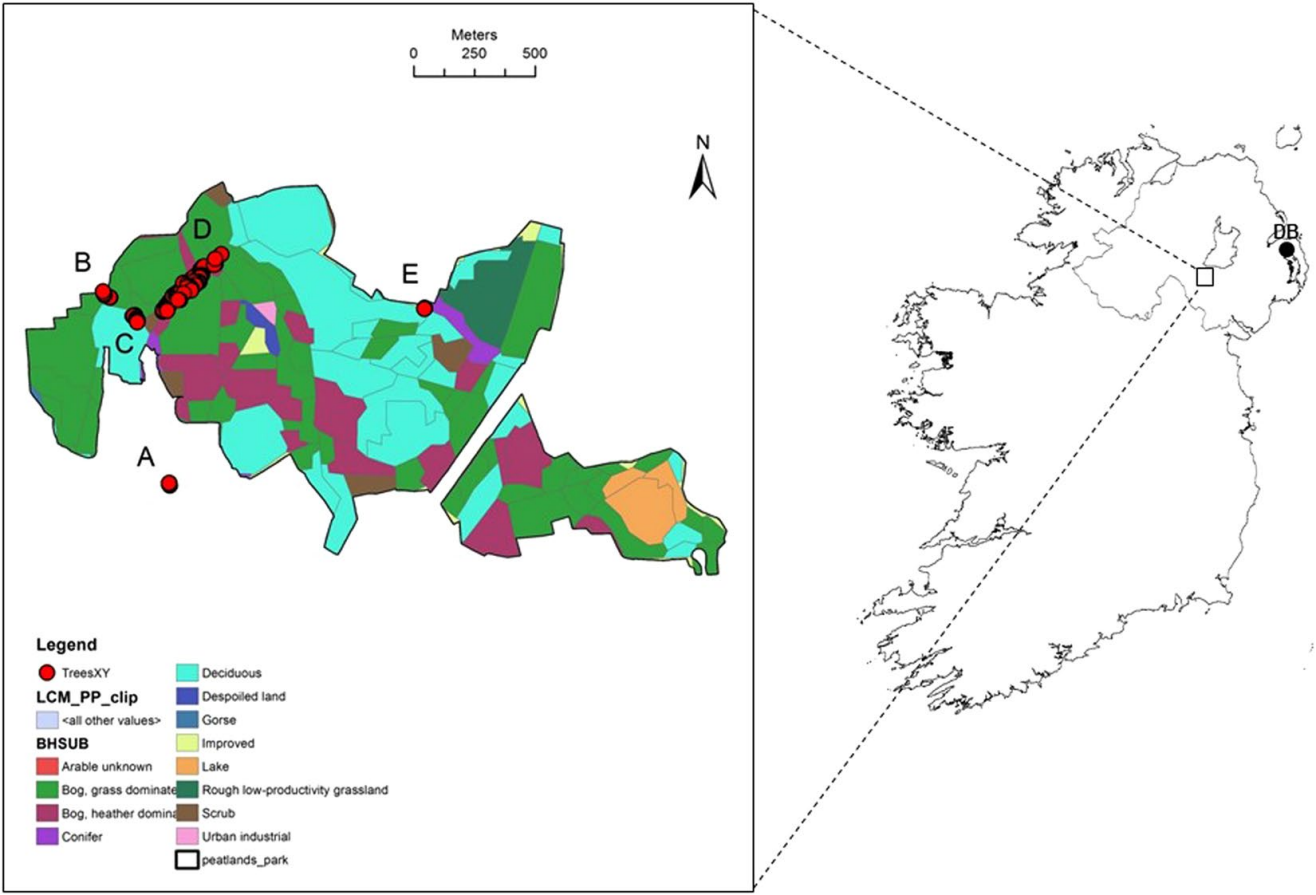
Location of the Peatlands Park population of *Frangula alnus* in Co. Armagh, Northern Ireland. Zoomed area shows the distribution of *F*. *alnus* in Peatlands Park, with the five fragments sampled (labeled A–E), mapped using ArcMap 10. The coloured area indicates a designated Special Area for Conservation (SAC). Land classes were taken from the CEH Land Cover Map (NERC/Centre for Ecology & Hydrology). DB – Drumawhey Bog (extinct population referred to in text).

*Frangula alnus* (alder buckthorn) is one of Ireland’s rarest tree species. Although widespread in temperate Europe, the species has a very limited and fragmented distribution in Ireland, where it has been in serious decline over the last few decades as a result of drainage of its preferred bogland habitat for alternative land use^10, 11^ (Fig. 1). In Northern Ireland, recent surveys suggested that *F*. *alnus* is restricted to the southern shores of Lough Neagh. Although there are records of the *F*. *alnus* previously occurring on the northern side of the Lough, as well as a single tree in Drumawhey Bog, County Down^12^, these are now extinct, the former natural woodland having been replaced by a broadleaf plantation^10^. The present-day surviving population has been part of Annagarriff Nature Reserve in Peatlands Park, County Tyrone since 1978, and the species is protected under the Wildlife (NI) Order (1985) and is a Priority Species for Conservation Action. The history of this population, which currently numbers *ca*. 140 individuals (see Methods and Fig. 1), is not well-documented. The earliest records mention “Twenty bushes on the NE margin of Annaghgarriff [sic] … before 1934”^10^, and census numbers appear to have remained low for many years, with a record of “about 30 young plants” around 1987–89^13^. Since then, there has been a gradual increase in numbers to those found today, possibly due to removal of rhododendron from the area, but it is not known whether the original trees were remnants of a once larger population, or whether numbers have always been low due to an initial founder effect. In recent years, the use of polymorphic microsatellite markers^14^ has allowed the testing of whether populations have gone through a bottleneck, based on theoretical expectations under mutation-drift equilibrium at a single point in time^15–18^. Consequently, the aim of the present study was to determine the levels of and patterns of genetic diversity in the remaining population to discover (1) whether there is any evidence for a genetic bottleneck, (2) if the establishment from a relatively limited number of individuals has been accompanied by a degree of inbreeding, and (3) whether there is any significant genetic substructuring within the population. In addition, as *F*. *alnus* is considered an invasive pest species in many countries^19, 20^, our findings could also shed light on the genetic demography of this recently expanded population with respect to similar invasive populations.

## Results

### Current distribution of *F*. *alnus* in Northern Ireland

Surveys of sites where *F*. *alnus* had been recorded previously found that the species is now restricted to a single location; Peatlands Park, Co. Armagh. The sole remaining population exists as five discrete clusters of plants numbering between 3–98 individuals, each separated by between 100–1,300 m (Fig. 1; Figure S1, Supplementary Material). In total, there are *ca*. 140 mature trees. The only other population recorded, at Drumawhey Bog on the northern edge of Strangford Lough, now appears to have been extirpated.

**Figure 2:**
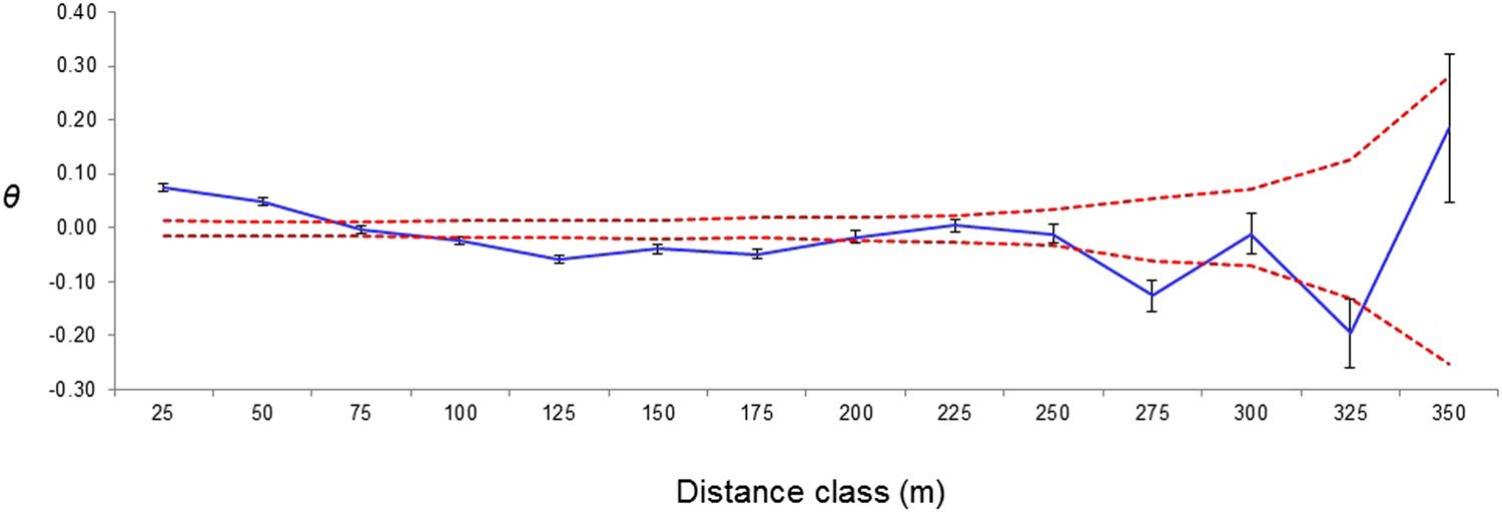
Correlogram of autocorrelation coefficient (*θ*; y-axis) plotted against distance (x-axis). 95% confidence intervals are indicated by dashed red lines.

According to the CEH Land Class Map 2007 and site visits, Subpopulation A resides in a suburban area and is the only subpopulation outside the SAC of Peatlands Park (Fig. 2), Subpopulation B resides in grass-dominated bog, Subpopulation C occurs on the edge of deciduous woodland, Subpopulation D is split between grass-dominated bog, heather-dominated bog and scrub, and Subpopulation E occurs on the boundary between a coniferous and deciduous woodland. The majority of the trees in the population occur in bog (40.1%), divided between grass dominated bog (17.4%) and heather dominated bog (22.7%). The next most common habitat type for this population is scrub (33.3%), then deciduous woodland (16.7%), suburban areas (8.3%) and the least common habitat type these trees occur in is coniferous woodland (1.5%).

### Levels of genetic diversity

No evidence of linkage disequilibrium was detected between the nine loci studied. Genotypes were obtained for 132 of the 139 trees (95%). Allele frequencies by locus and subpopulation are given in Table S1, Supplementary Material. Within-subpopulation levels of genetic diversity averaged across loci are given in Table 1 and ranged from 0.222 (Subpopulation E) to 0.404 (Subpopulation B) for observed heterozygosity (*H*_*O*_ mean = 0.314) and from 0.331 (Subpopulation C) to 0.423 (Subpopulation D) for expected heterozygosity (*H*_*E*_ mean = 0.387). Mean inbreeding coefficients (*F*_*IS*_) across loci (Table 1) ranged from −0.022 (Subpopulation C) to 0.625 (Subpopulation E; mean = 0.234). Three of the subpopulations had *F*_*IS*_ values significantly greater than zero. Diversity values and inbreeding coefficients calculated for Subpopulation E should be treated with some caution, as this fragment only contained three trees. Treating the five subpopulations as a single population gave values of 0.308, 0.411 and 0.251 for *H*_*O*_, *H*_*E*_ and *F*_*IS*_ respectively. Values of summary statistics by locus and subpopulation are given in Table S2, Supplementary Material. No evidence of a genetic bottleneck was detected under any of the three mutation models, with two of the nine nuclear loci studied showing a heterozygote excess under all three mutation models (Table 2).

**Table 1:** Diversity statistics.

Subpopulation	*N*	*H*_*O*_	*H*_*E*_	*F*_*IS*_
A	11	0.305	0.39	0.226**
B	6	0.404	0.422	0.050 NS
C	21	0.340	0.331	−0.022 NS
D	91	0.299	0.423	0.293**
E	3	0.222	0.372	0.625**
Mean	132	0.314	0.387	0.234
Total	132	0.308	0.411	0.251**

*N* – number of samples; *H*_*O*_ – observed heterozygosity; *H*_*E*_ – expected heterozygosity; *F*_*IS*_ – inbreeding coefficient. Significance of *F*_*IS*_: *P < 0.05; **P < 0.01; ***P < 0.001; NS – Not Significant.

**Table 2:** Results of the Bottleneck analysis.

	Locus								
	B101	A110	B7	A104	B106	B4	A7	A3	B9
Empirical data									
Sample size (haploid genomes)	262	244	264	264	262	230	264	256	258
Heterozygosity observed (*H*_*O*_)	0.482	0.713	0.067	0.269	0.407	0.348	0.593	0.244	0.578
Number of alleles observed (*k*_*O*_)	3	5	5	8	7	8	7	3	7
IAM									
Average heterozygosity (*H*_*E*_)	0.276	0.453	0.447	0.610	0.566	0.616	0.600	0.282	0.609
Standard deviation (SD)	0.188	0.182	0.180	0.141	0.157	0.136	0.145	0.189	0.150
Standard deviate ([*H*_*O*_ − *H*_*E*_]/SD)	**1.097**	**1.430**	−*2*.*113*	−*2*.*424*	−*1*.*010*	−*1*.*971*	−*0*.*046*	−*0*.*204*	−*0*.*210*
Probability (*H* > *H*_*E*_)	0.206	0.037	0.021	0.026	0.167	0.053	0.396	0.471	0.330
TPM									
Average heterozygosity (*H*_*E*_)	0.396	0.598	0.602	0.745	0.710	0.748	0.740	0.389	0.741
Standard deviation (SD)	0.166	0.115	0.118	0.070	0.077	0.065	0.071	0.161	0.074
Standard deviate ([*H*_*O*_ − *H*_*E*_]/SD)	**0.517**	**0.991**	−*4*.*516*	−*6*.*83*	−*3*.*966*	−*6*.*116*	−*2*.*064*	−*0*.*901*	−*2*.*218*
Probability (*H* > *H*_*E*_)	0.394	0.121	0.000	0.000	0.006	0.001	0.042	0.218	0.033
SMM									
Average heterozygosity (*H*_*E*_)	0.438	0.649	0.649	0.786	0.751	0.785	0.785	0.435	0.783
Standard deviation (SD)	0.137	0.089	0.093	0.047	0.058	0.048	0.048	0.144	0.052
Standard deviate ([*H*_*O*_ − *H*_*E*_]/SD)	**0.324**	**0.712**	−*6*.*268*	−*10*.*89*	−*5*.*902*	−*9*.*128*	−*4*.*206*	−*1*.*33*	−*3*.*968*
Probability (*H* > *H*_*E*_)	0.471	0.264	0.000	0.000	0.000	0.000	0.004	0.128	0.009

Values in bold indicate heterozygote excess. Values in italics indicate heterozygote deficiency.

**Figure 3:**
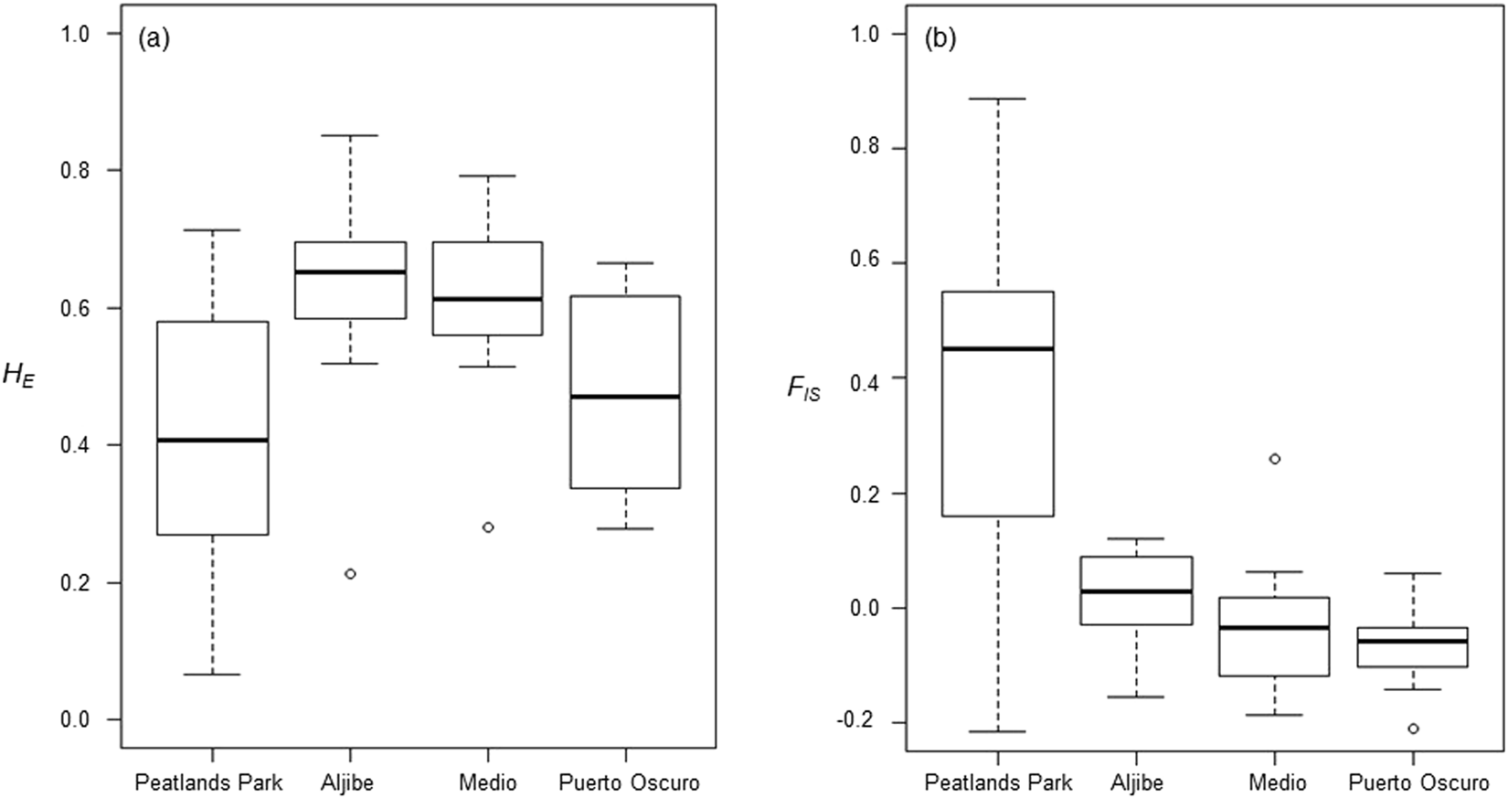
Boxplots showing values of (**a**) *H*_*E*_, and (**b**) *F*_*IS*_ in the Peatlands Park population analyzed in the present study and three Spanish populations analyzed in Riguiero *et al*. (2009).

The Peatlands Park population exhibited significantly lower levels of genetic diversity than two of the three Spanish populations based on the loci analyzed in the present study (*H*_*E*_ = 0.411 vs. *H*_*E*_ = 0.608 for Ajibe [Mann-Whitney test, *z* = −1.99, P_(2)_ = 0.047]; *H*_*E*_ = 0.603 for Medio [Mann-Whitney test, *z* = −1.99, P_(2)_ = 0.047]; *H*_*E*_ = 0.470 for Puerto Oscuro [Mann-Whitney test, *z* = −0.98, P_(2)_ = 0.327]; Fig. 3a). The mean value of *F*_*IS*_ for the Peatlands Park population (0.251) was significantly higher than those from the three Spanish populations (*F*_*IS*_ = 0.015 for Ajibe [Mann-Whitney test, *z* = 2.12, *P*_(2)_ = 0.034]; *F*_*IS*_ = −0.027 for Medio [Mann-Whitney test, *z* = 2.03, *P*_(2)_ = 0.042]; *F*_*IS*_ = −0.070 for Puerto Oscuro [Mann-Whitney test, *z* = 2.30, *P*_(2)_ = 0.021]; Fig. 3b).

No diversity was observed for the chloroplast microsatellites, with all six loci being monomorphic.

### Levels of genetic differentiation and spatial genetic structuring

Differentiation among subpopulations calculated from the analysis of molecular variance (AMOVA; Table 3) was *Φ*_*ST*_ = 0.0322 (*p* = 0.009). The BAPS analysis indicated that the first four subpopulations belonged to the same genetic cluster, with the fifth (Subpopulation E) being genetically distinct (Figure S2, Supplementary Material). The spatial autocorrelation analysis did, however, suggest that there may be some fine-scale spatial genetic structuring within Subpopulation D, with the autocorrelation coefficient being significantly higher than zero at the smallest scale (<50 m; Fig. 2).

**Table 3:** Analysis of molecular variance (AMOVA).

Source of variation	d.f	Variance components	% variation	Fixation index
Between subpopulations	4	0.05651	3.22	*Φ*_*ST*_ = 0.0322**
Within subpopulations	259	1.70113	96.78	

Significance of *Φ*_*ST*_: **P < 0.01.

## Discussion

The genetic analysis of the sole remaining population of *Frangula alnus* in Northern Ireland carried out in the present study suggests that establishment from a limited number of individuals has led to limited levels of genetic variation, accompanied by potential inbreeding during the recent expansion in census population size. A comparison with results from a study on *F*. *alnus* in Spain^21^ suggests that the Peatlands Park population has significantly lower levels of genetic diversity than two of the three Spanish populations. The complete lack of genetic variation in the six chloroplast microsatellite loci studied is also consistent with a founder effect. Although comparable data for these markers are not available for the Spanish populations, chloroplast microsatellites represent the most variable regions of the chloroplast genome^22^, and have also been shown to be monomorphic in a previously well-documented population bottleneck in Torrey pine^23^. The observed differences in levels of diversity between Northern Ireland and Spain could also reflect longer-term historical factors, since the Spanish populations most likely represent refugial populations during the last glaciation, and that the low levels observed in Northern Ireland are consistent with the founder effects associated with postglacial recolonization (“southern richness and northern purity”)^24, 25^. Interestingly, though, a further study on populations from Italy, France, Belgium and Sweden using 186 single nucleotide polymorphisms (SNPs) showed no decrease in genetic diversity with latitude^26^.

Despite the low levels of genetic variation, the Wilcoxon test for heterozygote excess did not indicate the occurrence of a bottleneck. Seven of the nine loci indicated a heterozygote deficiency, which could be indicative of population stability or constant growth^15^. Although there are no records predating 1934, evidence suggests that the population remained relatively stable from this time until the end of the 1980s, after which numbers increased to the current census of *ca*. 140 individuals^10, 13^. It may be unwise to take this at face value, though, since the power of the test is affected by several parameters, including number of loci used, number of generations since the bottleneck, the length of the bottleneck itself, and the magnitude of reduction in effective population size. Immigration, another potentially confounding factor, can most likely be ruled out, since the closest known *F*. *alnus* populations to that in Peatlands Park are found *ca*. 125 km away in Co. Westmeath, Ireland. Likewise, the mutation model is unlikely to have an effect, since the results were broadly consistent across the three models analyzed, and changing the parameters of the two-phase model from 90% single-stepwise mutations to 70% also had no effect. The generation time of *F*. *alnus* has been estimated to be between 5 years in Central Europe to around 20 years in the South^27^, so the maximum number of generations of growth is likely to be much less than 10, although the period of apparent population stability preceding this could be upwards of 10 generations. These time scales may limit the power of the heterozygote excess test to detect any possible bottleneck, but this may be balanced to some extent by the fact that, based on historical records, the effective population size (*N*_*e*_) after the bottleneck is unlikely to be any higher than *ca*. 20^15^.

Removal of rhododendron from areas of the park since the designation of the Annagarriff Nature Reserve may have facilitated the increase in numbers of *F*. *alnus* (Keith Stanfield, personal communication), but this has apparently been accompanied by a degree of inbreeding, based on *F*_*IS*_ values. Such inbreeding is most likely a result of the relatively limited genetic base of the population, particularly compared to the other populations of *F*. *alnus*. The mean value of *F*_*IS*_ for the Peatlands Park population is significantly higher than those from the three Spanish populations studied previously^21^. It is also far higher than the range of values reported in *F*. *alnus* populations across Europe based on SNPs (*F*_*IS*_ = −0.107–0.088, mean = −0.015)^26^. Reproductive dominance by a relatively low number of highly fecund individuals within a population could give rise to biparental inbreeding^28, 29^, but since *F*. *alnus* possesses a self-incompatibility mechanism, as indicated by crossing studies^30^, this would be dependent on the diversity of *S* alleles in the population. Previous studies have indicated a breakdown in self-incompatibility following population bottlenecks^31, 32^, but such a possibility would have to be tested via controlled pollination experiments.

The absence of high levels of spatial structuring of genetic variation in the Peatlands Park population of *F*. *alnus* indicates a general lack of barriers to dispersal, which is unsurprising given the small spatial scale relative to the potential dispersal distances of pollinators. The overall level of population differentiation (*Φ*_*ST*_ = 0.032) was lower than the average values for outcrossing species with seeds dispersed by gravity (0.152) or ingestion (0.200) quoted by Hamrick & Godt^33^, as well as the average value for biparentally inherited markers in angiosperms (0.184) quoted by Petit *et al*.^34^. The spatial autocorrelation analysis suggested some fine-scale genetic structure in Subpopulation D, probably due to limited seed dispersal. Seeds of *F*. *alnus* can be dispersed by birds^27, 35^, but in the closely related (congeneric in some classifications)^36^ *Rhamnus cathartica*, it has been shown that 90% of fruits fall beneath the mother tree^37^. This scenario could at least in part be responsible for the high *F*_*IS*_ observed in Subpopulation D, by way of a Wahlund effect.

The area surrounding Peatlands Park has a number of apple orchards with large numbers of wild bees in the vicinity, and the high incidence of fruits in the Peatlands Park *F*. *alnus* population suggests substantial levels of cross-pollination. Bees have large foraging ranges, even across sub-optimal habitats, and are likely to be important in maintaining the connectivity observed between the fragments in the present study^38, 39^. In contrast to the levels of fruiting found in Peatlands Park, where almost all trees had multiple fruits, a previous study on reproduction in populations of *F*. *alnus* from Cádiz, Spain found that only 2.8% of open-pollinated flowers set fruit^30^. Another study on southern range edge populations also indicated that the majority of ovule losses were due to cross-pollen limitation and extensive geitonogamy, and that seed output in the populations was limited to a few large trees^40^. The high percentage of fruiting trees observed in Peatlands Park suggests that this population is more similar to those found in Central European populations, which have a shorter generation time and higher levels of fruit production compared to Southern Iberian populations^27^. If the observed high *F*_*IS*_ values reflect some degree of inbreeding, there has been no apparent impact on fitness, at least in terms of fruit production.

Although the BAPS analysis assigned Subpopulation E to a separate genetic cluster from the other four subpopulations, this should be taken with some degree of caution for several reasons. Firstly, the subpopulation numbers only three individuals, and thus allelic frequencies will be skewed. Secondly, it has been shown previously that the BAPS algorithm tends to over-estimate the true number of genetic clusters present in the data^41^. Finally, only a single private allele is present in Subpopulation E, with the majority of genetic differentiation being due to the aforementioned differences in allele frequencies at several of the loci studied.

Whilst it is true that knowledge of the evolutionary dynamics of natural populations with respect to demography and gene flow allows the management of threatened plant populations to go beyond simple “conservation gardening”^42^, in the case of *F*. *alnus* in Northern Ireland it would appear that edaphic and ecological factors are of greater importance. The land classes that are correlated with occurrence of alder buckthorn include bog, conifer and pastures, and this is consistent with the literature that claims this species likes open lowland areas with moist, fertile soils^43, 44^. Nevertheless, there are large areas of apparently suitable habitat in Peatlands Park which have not been colonized during the recent expansion of the species in the nature reserve. This would suggest that although the low levels of genetic diversity and potential inbreeding revealed in the population are of some concern with respect to evolutionary potential, more immediate conservation efforts might be better focused on ensuring suitable habitat for the continued recovery of this isolated population. Additionally, consideration should be given to possible supplementation using material from the populations in the Republic of Ireland, which are larger and may harbor additional genetic diversity.

## Methods

### Study species

*Frangula alnus* Miller (syn. *Rhamnus frangula* L.) is a small tree or shrub found across temperate Europe from northern Scandinavia and Russia to the Mediterranean, where it is comparatively scarce^43^. In Britain and Ireland, the species tends to be found in damp, boggy habitats, but can also colonize drier ground outside of its native range, particularly in North America where the species is now considered an invasive alien^19, 20, 35^. Reproduction in *F. alnus* is exclusively sexual^30^. Flowers are hermaphroditic and well-adapted to insect pollination as they have nectar, colour and odour. They are generalised entomophiles, with orders Hymenoptera, Diptera and Coleoptera providing the main pollinators, and occasional pollination by Lepidoptera^40^. Controlled pollination studies revealed almost no selfing or geitonogamy, indicating the existence of self-incompatibility mechanisms, although occasional selfing in the absence of insect pollinators has been reported^30^. Field studies suggest that limited fruit initiation is primarily due to low levels of cross-pollination^30, 40^. Seeds are dispersed either by gravity or via ingestion by birds^27, 35, 37^.

### Surveys and sampling

Surveys were carried out across Northern Ireland in the summer of 2007 at sites where *F. alnus* had been found previously based on records from the National Biodiversity Network (NBN) Gateway (http://data.nbn.org.uk) and the Centre for Environmental Data and Recording (CEDaR: http://www.habitas.org.uk/cedar/). Frangula alnus is restricted to the southern shores of Lough Neagh, where it exists as a fragmented population from a single location in Annagarriff Nature Reserve in Peatlands Park, County Tyrone (Fig. 1). All mature trees were numerically tagged with metal tags, GPS coordinates recorded, and a leaf sample from each obtained for genetic analysis. The GPS coordinates were loaded into ESRI ArcMap 10 and plotted on top of a CEH Land Cover Map 2007^45^ to discover the main land class that the trees occupy. DNA was extracted from leaf material using the CTAB method^46^. Genotypes were successfully obtained for 132 of the 139 trees sampled.

### Nuclear microsatellite analysis

We attempted to genotype all mature plants for sixteen previously described microsatellite loci for *F*. *alnus* ^21^. Of these, four (FaA103, FaA125, FaA8 and FaB8) could not be consistently amplified, and three (FaA12, FaB102 and FaA116) were monomorphic, leaving nine polymorphic loci: FaB101, FaA110, FaB7, FaA104, FaB106, FaB4, FaA7, FaA3 and FaB9. All reactions were carried out on a MWG Primus thermal cycler. PCR was carried out in a total volume of 10 μl containing 100 ng genomic DNA, 10 pmol of dye-labelled forward primer (HEX), 1 pmol of tailed forward primer, 10 pmol reverse primer, 1x PCR reaction buffer, 200 μM each dNTP, 2.5 mM MgCl_2_ and 0.25 U GoTaq Flexi DNA polymerase (Promega). PCR conditions were as described previously^19^. Genotyping was carried out on an AB3730xl capillary genotyping system. Allele sizes were scored using the GeneMapper software package (V5.0; Applied Biosystems) and LIZ-500 size standards, and were checked by comparison with previously sized control samples.

### Chloroplast microsatellite analysis

Chloroplast microsatellite markers were developed by identifying mononucleotide regions of ten or more repeats in partial *F. alnus* chloroplast genome sequences either from GenBank or in regions amplified and sequenced *de novo* using universal chloroplast primers^22^. The *trn*T*-trn*F, *atp*H-*atp*I, *atp*I-*rpo*C2, *rps*18-*clpp*, *psb*C-*trn*S, *trn*S-*trnf*M and *rpl*16-*rps*3 (UCP6) regions were amplified and sequenced using the primers described in Grivet *et al*.^47^ and Provan *et al*.^48^. Species-specific primers were designed using the Primer3 program to amplify six chloroplast microsatellites (Table S3, Supplementary Material). Primers were tailed as described previously, and PCR was carried out in 10 μl reactions as described previously using the following conditions: initial denaturation at 94 °C for 3 min followed by 30 cycles of denaturation at 94 °C for 30 s, annealing at 56 °C for 30 s, extension at 72 °C for 30 s and a final extension at 72 °C for 5 min. Genotyping was carried out as described previously.

### Data analysis

Subpopulations of the Peatlands Park population were classed as distinct groups of trees with no other trees of this species growing within 100 m between groups, resulting in five distinct subpopulations (Fig. 1). GenePop V3.4^49^ was used to test for linkage disequilibrium between nuclear loci. To estimate genetic diversity within the population, levels of observed (*H*_*O*_) and expected (*H*_*E*_) heterozygosity, and fixation indices (*F*_*IS*_) were calculated using the Arlequin (V3.5.1.2)^50^ and Fstat (V2.9.3.2)^51^ software packages respectively. Significance of *F*_*IS*_ was determined by 10,000 randomization steps. To test for the occurrence of a genetic bottleneck, the Wilcoxon test for heterozygote excess was performed under the infinite alleles model (IAM), the stepwise mutation model (SMM) and a two-phase model (TPM) incorporating 90% single-stepwise mutations using the program Bottleneck (V1.2)^52^. The Wilcoxon test was used as it is recommended for a relatively low number of loci.

To compare genetic diversity between the population analyzed in the present study, and those from three Spanish populations previously analyzed using the same nuclear microsatellites^21^, mean values for *H*_*E*_ and *F*_*IS*_ were calculated over the twelve loci successfully amplified in the present study, including the three loci which were monomorphic. Mann-Whitney tests were carried out to assess the significance of differences in *H*_*E*_ and *F*_*IS*_.

The level of genetic differentiation between the five fragments was estimated using *Φ*_*ST*_, which gives an analogue of *F*_*ST*_^53^ calculated within the analysis of molecular variance (AMOVA) framework^54^ using Arlequin. To identify possible spatial patterns of gene flow, the software package BAPS (V5)^55^ was used to identify clusters of genetically similar subpopulations using a Bayesian approach. Ten replicates were run for all possible values of the maximum number of clusters (*K*) up to *K* = 5, the number of subpopulations, with a burn-in period of 10 000 iterations followed by 50 000 iterations. Multiple independent runs always gave the same outcome. To further identify possible spatial patterns of gene flow, spatial autocorrelation analysis was carried out for Subpopulation D, the largest of the five subpopulations, using SPAGeDi (V1.4)^56^. Mean coancestry coefficients (*θ*_*xy*_)^57^ between pairs of individuals were calculated at 25 m distance class intervals, and plotted as a correlogram, with 95% confidence intervals calculated from 1,000 permutations of individuals within each distance class, and for estimates of *θ*_*xy*_ using 1,000 permutations.

## Electronic supplementary material

Supplementary Info
