# Supplementary material for: Low genetic diversity and potential inbreeding in an isolated population of alder buckthorn (Frangula alnus) following a founder effect
Source: Sci Rep. 2017 Jun 7;7:3010. doi: 10.1038/s41598-017-03166-1 (PMC5462792; doi:10.1038/s41598-017-03166-1)
Supplement: Supplementary file 1 — Supplementary Info [file 41598_2017_3166_MOESM1_ESM.pdf]

Supplementary Information

"Low genetic diversity and potential inbreeding in an isolated population of alder buckthorn (*Frangula alnus*) following a founder effect"

Caroline MV Finlay, Caroline R Bradley, S Jane Preston, Jim Provan

Table S1 Allele frequencies by locus and subpopulation.

Table S2 Diversity statistics for each locus by subpopulation.

Table S3 *Frangula alnus* chloroplast microsatellite primers.

Figure S1 Map showing locations of individual trees.

Figure S2 Results of the BAPS analysis.

Table S1 Allele frequencies by locus and subpopulation.

Locus	Allele	Subpopulation				
		A	B	C	D	E
FaB101	232	0.650	0.500	0.571	0.605	1.000
	236	0.350	0.500	0.429	0.390	
	240				0.005	
FaA110	315				0.006	
	317	0.200	0.125	0.300	0.218	0.500
	319	0.100	0.125	0.275	0.149	
	321	0.550	0.375	0.375	0.420	0.500
	329	0.150	0.375	0.050	0.207	
FaB7	122	0.045				
	124				0.011	
	126	0.955	1.000	1.000	0.967	0.667
	128					0.033
	138				0.022	
FaA104	172				0.011	0.333
	174				0.005	
	176				0.005	
	178	0.864	0.750	0.929	0.841	0.667
	190				0.011	
	192	0.136	0.250	0.071	0.104	
	194				0.011	
	200				0.011	
FaB106	223				0.011	
	225			0.238	0.211	
	229		0.167		0.039	0.167
	231	0.091				
	239	0.919	0.833	0.762	0.722	0.666
	241				0.006	0.167
	247				0.011	
FaB4	279				0.039	
	281	0.150	0.167	0.225	0.072	
	283	0.850	0.833	0.775	0.796	0.833
	287				0.013	
	293				0.013	
	297				0.046	0.167
	301				0.007	
	305				0.013	
FaA7	150		0.167			
	160	0.091			0.011	
	164	0.409	0.033	0.429	0.242	
	178		0.083	0.048	0.049	
	184	0.136			0.032	
	186	0.364	0.033	0.476	0.615	1.000
	200		0.083	0.048	0.049	
FaA3	288	0.100			0.140	
	294	0.900	1.000	1.000	0.815	1.000
	300				0.045	
FaB9	284	0.273		0.071	0.056	
	290				0.006	
	292	0.227	0.500	0.048	0.270	0.833
	296		0.100			
	298	0.045				
	300			0.048	0.079	
	302	0.455	0.400	0.833	0.590	0.167

Table S2 Diversity statistics for each locus by subpopulation. Abbreviations: H_O, observed heterozygosity; H_E, expected heterozygosity; F_{IS}, inbreeding coefficient. Significance of F_{IS} - * $P < 0.05$; ** $P < 0.01$; NS – non-significant.

Subpopulation	Locus								
	FaB101	FaA110	FaB7	FaA104	FaB106	FaB4	FaA7	FaA3	FaB9
A	H_O = 0.500	H_O = 0.600	H_O = 0.091	H_O = 0.273	H_O = 0.000	H_O = 0.100	H_O = 1.000	H_O = 0.000	H_O = 0.182
	H_E = 0.479	H_E = 0.658	H_E = 0.091	H_E = 0.247	H_E = 0.173	H_E = 0.268	H_E = 0.706	H_E = 0.189	H_E = 0.697
	F_{IS} = -0.047^{NS}	F_{IS} = 0.092^{NS}	F_{IS} = 0.000^{NS}	F_{IS} = -0.111^{NS}	F_{IS} = 1.000*	F_{IS} = 0.640^{NS}	F_{IS} = -0.447^{NS}	F_{IS} = 1.000^{NS}	F_{IS} = 0.748**
B	H_O = 0.667	H_O = 0.500	H_O = 0.000	H_O = 0.500	H_O = 0.000	H_O = 0.333	H_O = 0.833	H_O = 0.000	H_O = 0.800
	H_E = 0.545	H_E = 0.786	H_E = 0.000	H_E = 0.409	H_E = 0.303	H_E = 0.303	H_E = 0.803	H_E = 0.000	H_E = 0.644
	F_{IS} = -0.250^{NS}	F_{IS} = 0.400^{NS}	F_{IS} = N/A	F_{IS} = -0.250 ^{NS}	F_{IS} = 1.000^{NS}	F_{IS} = -0.111^{NS}	F_{IS} = -0.042^{NS}	F_{IS} = N/A	F_{IS} = -0.280^{NS}
C	H_O = 0.762	H_O = 0.750	H_O = 0.000	H_O = 0.143	H_O = 0.095	H_O = 0.150	H_O = 0.952	H_O = 0.000	H_O = 0.190
	H_E = 0.502	H_E = 0.709	H_E = 0.000	H_E = 0.136	H_E = 0.372	H_E = 0.358	H_E = 0.599	H_E = 0.000	H_E = 0.303
	F_{IS} = -0.538^{NS}	F_{IS} = -0.059^{NS}	F_{IS} = N/A	F_{IS} = -0.053^{NS}	F_{IS} = 0.748**	F_{IS} = 0.587*	F_{IS} = -0.613^{NS}	F_{IS} = N/A	F_{IS} = 0.377*
D	H_O = 0.472	H_O = 0.563	H_O = 0.000	H_O = 0.197	H_O = 0.233	H_O = 0.197	H_O = 0.648	H_O = 0.169	H_O = 0.213
	H_E = 0.485	H_E = 0.715	H_E = 0.065	H_E = 0.283	H_E = 0.434	H_E = 0.359	H_E = 0.560	H_E = 0.316	H_E = 0.586
	F_{IS} = 0.026^{NS}	F_{IS} = 0.214**	F_{IS} = 1.000**	F_{IS} = 0.303**	F_{IS} = 0.464**	F_{IS} = 0.452**	F_{IS} = -0.159^{NS}	F_{IS} = 0.469**	F_{IS} = 0.637**
E	H_O = 0.000	H_O = 1.000	H_O = 0.000	H_O = 0.000	H_O = 0.333	H_O = 0.333	H_O = 0.000	H_O = 0.000	H_O = 0.333
	H_E = 0.000	H_E = 1.000	H_E = 0.533	H_E = 0.533	H_E = 0.600	H_E = 0.333	H_E = 0.000	H_E = 0.000	H_E = 0.333
	F_{IS} = N/A	F_{IS} = N/A	F_{IS} = 1.000^{NS}	F_{IS} = 1.000^{NS}	F_{IS} = 0.500^{NS}	F_{IS} = 0.000^{NS}	F_{IS} = N/A	F_{IS} = N/A	F_{IS} = 0.000^{NS}

Table S3 *Frangula alnus* chloroplast microsatellite primers.

Locus	Repeat	Region	Primers	Size
FaCPSSR1	$(T)_{10}$	*trn*L-*trn*F intergenic	CCAAACCAAATGATTAATGACG<br>ATATTCGATTCTGTTTTCAACTTGG	126 bp
FaCPSSR2	$(A)_{10}$	*atp*I-*rpo*C2 intergenic	TCAATGGTCAATCTCCGGTA<br>TTCTCCCCACACTTCTTATCG	146 bp
FaCPSSR3	$(A)_{11}$	*trn*T-*trn*D intergenic	TTTTTCTTTATTTTACTCTTTTCCTTAGA<br>ATATATCTCTACTGCTATTTATTTACTC	154 bp
FaCPSSR4	$(A)_{11}$	*trn*H-*trn*K intergenic	TGTCAAACCATTCACCTTTTT<br>CCTTTCTTTTACTTTTATATTTATATATGTTCTTC	172 bp
EU749835	$(T)_{10}$	*psb*K-*psb*I intergenic	GAAAAACTCCACTTTTATTACAAATATTACA<br>AACACCAAGAACGAAGGTTTT	118 bp
EU750523	$(A)_{11}$	*trn*H-*psb*A intergenic	AAGATGAAATACAAGATAAAAGATAATGG<br>TCAACTAATTCTTCGTTATGTAGAAACC	176 bp

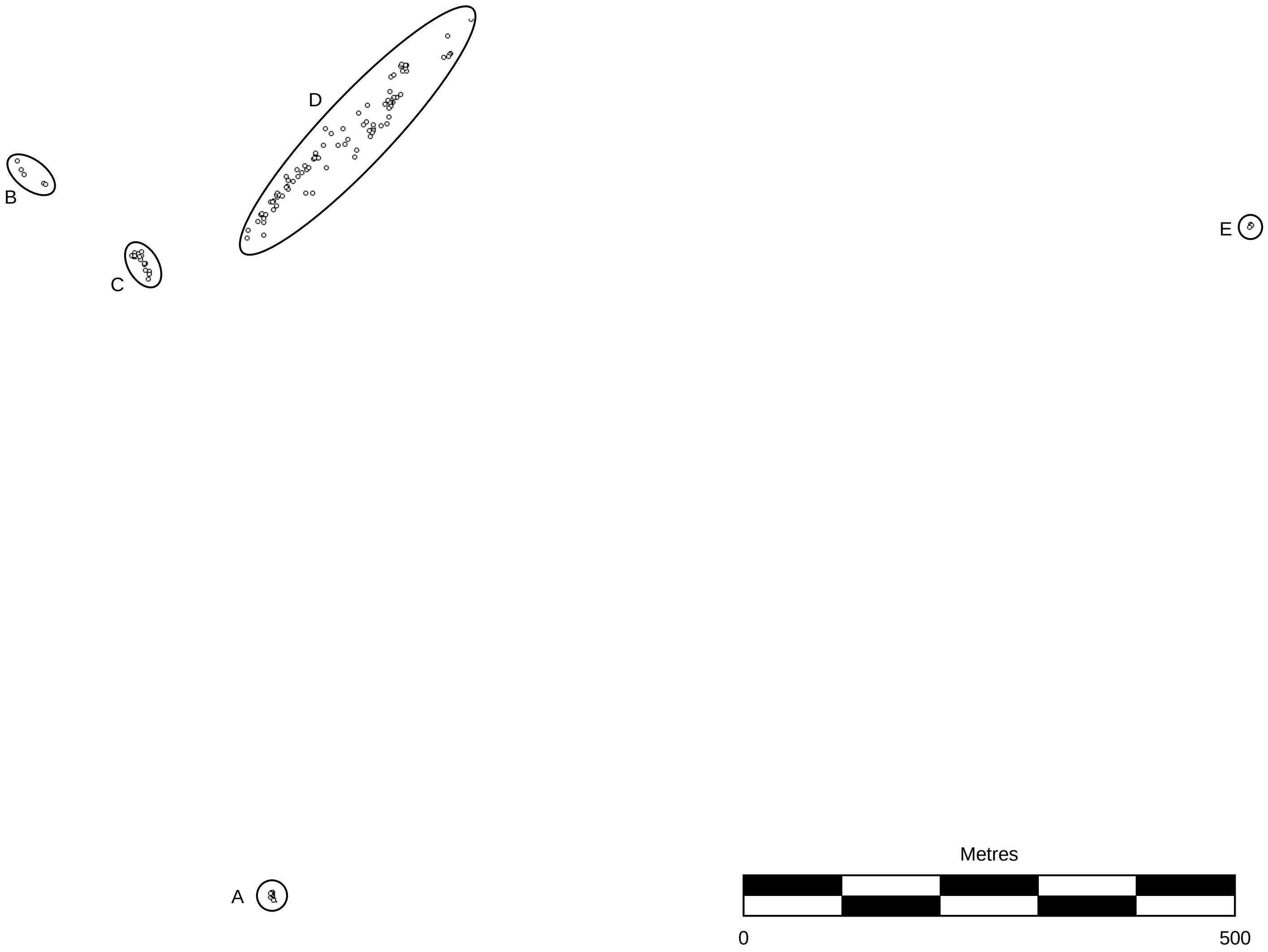
D
B
C
E
A
Metres
0
500

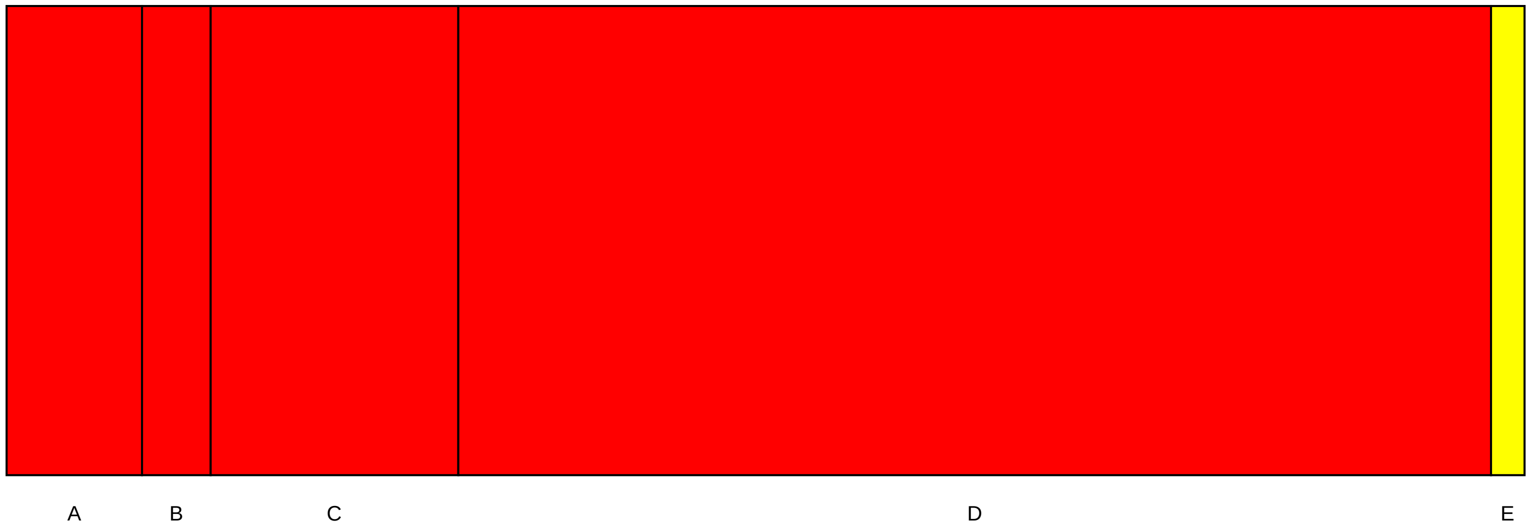
A
B
C
D
E
